# Splicing modulator FR901464 is a potential agent for colorectal cancer in combination therapy

**DOI:** 10.18632/oncotarget.26564

**Published:** 2019-01-08

**Authors:** Tomoki Yamano, Shuji Kubo, Aya Yano, Tomoko Kominato, Shino Tanaka, Masataka Ikeda, Naohiro Tomita

**Affiliations:** ^1^ Division of Lower Gastrointestinal Surgery, Hyogo College of Medicine, Nishinomiya, Hyogo, Japan; ^2^ Laboratory of Molecular and Genetic Therapeutics, Institute for Advanced Medical Sciences, Hyogo College of Medicine, Nishinomiya, Hyogo, Japan

**Keywords:** SF3B1, splicing, modulator, colorectal cancer, combination

## Abstract

FR901464 (FR) was first described as an anticancer drug and later identified as a modulator of splicing factor 3B subunit 1 (SF3B1). Although the effectiveness of splicing modulators has been investigated in colorectal cancer (CRC) cells, their usefulness in animal experiments has not been confirmed. The association of *SF3B1* with CRC progression and the influence of FR on transcriptional activity in CRC has not been fully elucidated. FR showed strong cytotoxicity against CRC cell lines, *SF3B1*-mutated cancer cell lines, and human fibroblasts with IC_50_ values less than 1 ng/ml. FR-resistant clones derived from HCT116, DLD1, Lovo, and CT26 cells showed IC_50_ values greater than 100 ng/ml. *SF3B1* sequencing demonstrated low frequencies of *SF3B1* mutations in CRC and mutations in codon 1074 of exon 22 in all FR-resistant clones. Unlike hematological malignancies, *SF3B1* expression was not associated with CRC progression. Although FR showed significant growth inhibition in a xenograft model of RKO cells, severe toxicity was also induced. These data indicated CRC might be a suitable target of FR unless toxicity occurs. Microarray analysis and real-time quantitative PCR demonstrated downregulation of genes associated with Fanconi anemia (*BRCA1 and BRCA2*) and 28 driver oncogenes. These data suggested combination treatment of FR with other anticancer drugs whose sensitivity is associated with genes affected by FR treatment. Combination treatment with PARP1 inhibitor olaparib, whose sensitivity was enhanced by *BRCA* 1/2 deficiency, showed synergistic effects in CRC cells. Our data indicates the potential of FR in combination therapy rather than monotherapy for CRC treatment.

## INTRODUCTION

FR901464 (FR) and FR901228 were discovered as anticancer drugs by Nakajima et al. [1–3] and were subsequently identified as a SF3B1 inhibitor and a histone deacetylase inhibitor (HDACI), respectively [4, 5]. FR901228 and other HDACIs with different chemical structures have similar biological effects on cancer cells, including induction of cell cycle arrest, differentiation, and apoptosis, by influencing transcriptional activity [6, 7]. Because FR was also shown to enhance the transcriptional activity of SV40 promoter [1] we postulated that FR might also have biological effects in cancer cells by influencing transcriptional activity.

In 2007, FR, its methylated derivative Spliceostatin A (SSA), and Pladienolides were identified as inhibitors of splicing factor 3B subunit 1 (SF3B1), a critical spliceosome component that plays a role in the synthesis of mature mRNA by editing pre-mRNA molecules [5, 8]. Since then, Pladienolides and FR analogues including SSA and Meayamaicin have been investigated as anticancer drugs by modulating splicing through targeting the SF3B1 subunit of the spliceosome [9–24].

In 2011, *SF3B1* mutations were identified in hematological malignancies including myelodysplastic syndromes (MDS) and chronic lymphocytic leukemia (CLL) using next-generation sequencing [25–30]. In MDS, *SF3B1* mutations are present at frequencies of 20%−30% and are associated with the presence of ring sideroblasts and with favorable clinical outcomes and a lower incidence of leukemia [25–27]. In CLL, *SF3B1* mutations are present in approximately 10%−15% of patients and are associated with accelerated disease progression and poor survival [28–30]. Some solid tumors, including uveal melanoma [31–33], breast cancer [34, 35], and pancreatic cancer [36], have relatively low frequencies of *SF3B1* mutations compared with hematological malignancies. In uveal melanoma, *SF3B1* mutations are associated with alternative splicing and indicate a good prognosis [33]. These mutations were identified between exon 10 and exon 16, including at mutational hot spots between exons 12 and 15 [26].

Colorectal cancer (CRC) is one of the most common malignancies in Western countries and Japan [37]. Advanced metastatic CRC is still a lethal disease despite the recent application of combination chemotherapy involving 5-fluorouracil (5-FU) and oxaliplatin (OHP) or irinotecan with molecular targeted therapy against vascular endothelial growth factor (VEGF) and epidermal growth factor receptor (EGFR) [38].

Yokoi et al. and Teng et al. reported *SF3B1* mutations at codon 1074 (exon 22) in CRC cells resistant to Pladienolides [16, 23]. CRC cell lines have been widely used for the study of splicing modulators [11, 13–15, 18, 22]. However, the development of splicing modulators for CRC treatment has been limited and the influence of SF3B1 on CRC progression was not determined.

In the present study, we assessed the possible application of the splicing modulator FR for CRC treatment. First, we evaluated the cytotoxic effect of FR on CRC *in vitro* and *in vivo*. Next, we analyzed *SF3B1* mutations and expression in CRC cell lines and CRC tumors to assess the association between *SF3B1* and CRC progression. Then, the influence of FR on transcriptional activity and alternative splicing was evaluated. Finally, based on the influence of FR on *BRCA1* and *BRCA2*, we evaluated combination treatment with FR and the PARP1 inhibitor olaparib in CRC cells to explore the possible application of FR with other anticancer drugs.

## RESULTS

### Sensitivity of cancer cell lines to FR901464

**Figure 1:**
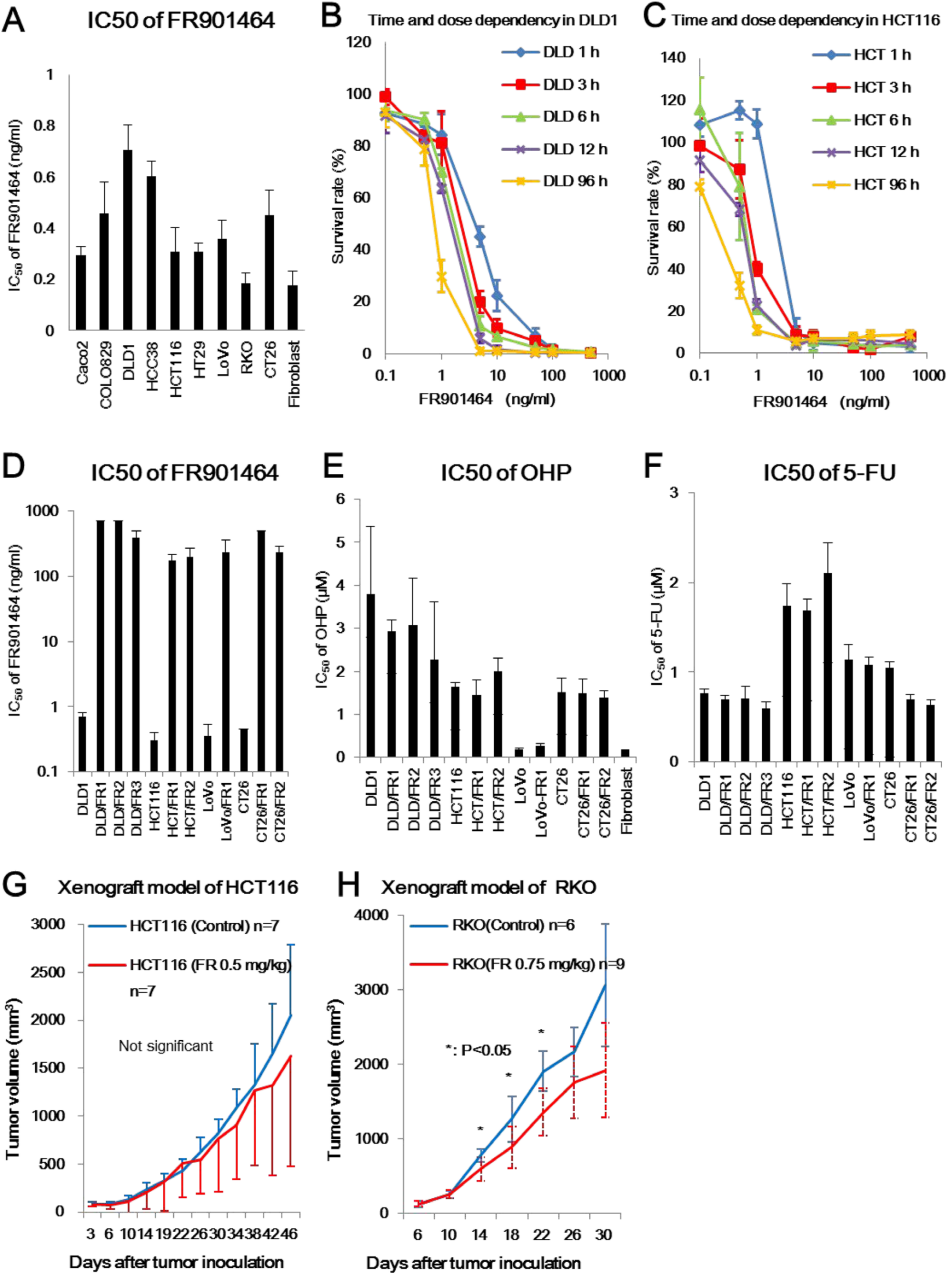
Cytotoxic effect of FR901464 *in vitro* and *in vivo* (**A**) IC_50_ values of FR in cancer cell lines. The IC_50_ values for FR in cancer cell lines and human fibroblasts were less than 1 ng/ml (range, 0.18 to 0.71 ng/ml). Bars represent the mean IC_50_ values with standard deviations. (**B, C**) Time- and dose-dependency of FR cytotoxicity in DLD1 cells (B) and HCT116 cells (C). FR showed cytotoxicity after 1 h of treatment in both cell lines. Treatment with FR for 3 h and 6 h induced similar cytotoxicity as 12 h of treatment in HCT116 and DLD1 cells, respectively, using Dunnett’s test. (**D, E, F**) IC_50_ values of FR, oxaliplatin (OHP), and 5-fluorouracil (5-FU) in parental and FR-resistant cells. FR-resistant clones showed significantly higher IC_50_ values of FR than their parental cells (*P* < 0.0001) (D). FR-resistant clones had the same IC_50_ values for OHP (E) and 5-FU (F) as the parental cells. Bars represent mean IC_50_ values with standard deviations. (**G, H**) Cytotoxicity of FR *in vivo*. Tumor growth was compared between xenograft models of HCT116 (G) or RKO (H) with or without intraperitoneal injection of FR. There was a significant difference in tumor growth between mice with and without FR treatment for RKO xenografts, but not for HCT116.

The IC_50_ values for FR in cancer cell lines and human fibroblasts were less than 1 ng/ml (0.18 to 0.71 ng/ml) (Figure 1A). The IC_50_ of DLD1 (0.71 ng/ml) was highest among the cell lines tested and was significantly higher than that of all other cell lines except for HCC38. The IC_50_ for HCT116 (0.31 ng/ml) was similar to that of other cells except for DLD1 and HCC38. The IC_50_ for human fibroblasts (0.18 ng/ml) was the lowest but statistically similar to that of other cell lines except for DLD1, HCC38, and COLO829. The melanoma cell line COLO829 and the breast cancer cell line HCC38, which were reported to have *SF3B1* missense mutations in exons 12 and 15, respectively, are also sensitive to FR [26].

The cytotoxicity of FR depended on the time and dose of treatment (Figure 1B and Figure 1C). Treatment with FR for 3 h and 6 h induced similar cytotoxicity to 12 h of treatment in HCT116 and DLD1 cells, respectively, using Dunnett’s test.

### Drug sensitivity of FR-resistant clones

DLD/FR1, DLD/FR2, DLD/FR3, HCT/FR1, HCT/FR2, LoVo/FR1, LoVo/FR2, LoVo/FR3, and LoVo/FR4 cell lines were derived from three cell lines (DLD1, HCT116, and LoVo) bearing microsatellite instability (MSI) and CT26/FR1 and CT26/FR2 cell lines were derived from CT26 cells with microsatellite stability (MSS) [39, 40]. IC_50_ values for FR were 173 ng/ml in HCT/FR1, 200 ng/ml in HCT/FR2, 389 ng/ml in DLD/FR3, 236 ng/ml in LoVo/FR1, and >500 ng/ml in DLD/FR1 and DLD/FR2 cells (Figure 1D). IC_50_ values of FR-resistant clones were significantly higher than those of their parental cells (*P* < 0.0001). In contrast, there were no significant differences in IC_50_ values of OHP (Figure 1E) or 5-FU (Figure 1F) between the parental cells and their FR-resistant clones. These data suggest that the mechanisms of FR resistance in these clones did not involve cross-resistance mediated by multidrug resistance genes. Human fibroblasts were sensitive to FR and OHP as well as the other cancer cell lines.

### Effect of FR on tumor growth

In mouse subcutaneous xenograft models, the antitumor effect of FR was accompanied by high toxicity. Three of seven mice receiving 0.5 mg/kg FR (Figure 1G) and four of nine mice receiving 0.75 mg/kg FR (Figure 1H) died following intraperitoneal administration of FR. However, there was significant inhibition of tumor growth in a xenograft model of RKO at day 14 (*P* = 0.038), day 18 (*P* = 0.04), and day 22 (*P* = 0.0086) (Figure 1H). The lack of a significant effect of FR on HCT116-derived xenografts (Figure 1G) was consistent with results of a previous study using FR901464 analogues [13].

### Sequencing of *SF3B1* in cancer cell lines and CRC patients

Sanger sequencing demonstrated that all FR-resistant cell lines carried missense mutations in codon 1074 of *SF3B1* (Table 1). This codon has been reported to be associated with drug resistance to other splicing modulators [16, 23]. Two types of mutation were present in the human cells: CGT to TGT (Arg1074Cys) in DLD1/FR1, DLD/FR2, DLD/FR3, and LoVo/FR2 cells; and CGT to CAT (Arg1074His) in HCT/FR1, HCT/FR2, LoVo/FR1, LoVo/FR3, and LoVo/FR4 cells (Table 1). We also detected nonsense mutations in *SF3B1* exon 10 in DLD1 (Arg451Stop) and LoVo-FR1 (Gln459Stop). Murine CT26 cells contained the missense mutation CGA to CAA (Arg1074Gln) in CT26/FR1 and CGA to ATT (Arg1074Ile) due to insertion of AAA in codon 1070 in CT26/FR2.

**Table 1:** Details of SF3B1 mutations

Cell / Exon(codon)	10(451, 459)	22(1074)
DLD1	(451) CGA(R)→TGA(end)	–
DLD/FR1, DLD/FR2, DLD/FR3	(451) CGA(R)→TGA(end)	CGT(R)→TGT(C)
HCT/FR1, HCT/FR2	–	CGT(R)→CAT(H)
LoVo/FR1	(459) CAG(Q)→TAG(end)	CGT(R)→CAT(H)
LoVo/FR4, LoVo/FR5	–	CGT(R)→TGT(C)
LoVo/FR3	–	CGT(R)→CAT(H)
CT26/FR1	– –	CGA(R)→CAA(G) CGA(R)→ATT(I)
CT26/FR2		

All human FR-resistant clones were derived from human CRC cells with MSI, which are known to carry many gene mutations [38]. We sequenced all of the *SF3B1* exons in 50 patients with MSS (45 CRC and five other tumors) and in 31 patients with MSI (30 with CRC and one with small bowel gastrointestinal stromal tumor) but no mutations were detected in these 81 patients (Table 2).

**Table 2:** Background of samples (number of samples with mutation/number of samples analyzed)

Exons analyzed		1–25	8, 10, 12–15, 22	8, 10, 22	Total
CRC cell lines		1/6 (exon 10)	–	–	1/6
CRC tumors		0/75	0/66	0/105	0/246
	MSS	45	66	105	
	MSI	30	0	0	
Other Tumors		0/6	–	0/1	0/7
	MSS	5		1	
	MSI	1		0	
**Total**		1/87	0/66	0/106	1/259

Abbreviations: CRC; colorectal cancer; MSS, microsatellite stable, MSI; microsatellite instable.

All human FR-resistant clones and their parental cells had silent mutations in *SF3B1* exon 8 (codon 320 in LoVo and codon 371 in DLD1 and HCT116), whereas the other samples had no exon 8 mutations. Therefore, we postulated that mutations in exon 8 could be used to predict the possibility of *SF3B1* exon 10 or exon 22 mutations. We further assessed samples from 171 CRC tumors and one metastatic renal cancer in the colon and found no *SF3B1* mutation in exons 8, 10, and 22 (Table 2). Of the 171 CRC tumors, we found 66 CRC tumors bore no *SF3B1* mutation in exons 12−15, which were recognized as mutational hotspots in other malignancies [26].

We found no *SF3B1* mutations in these 253 tumors including 246 CRC tumors (Table 2). These data suggest that *SF3B1* mutations are rare in malignancies of the lower gastrointestinal tract irrespective of MSI or MSS status.

### Influence of FR, *SF3B1* mutation, and CRC progression on *SF3B1* expression

**Figure 2:**
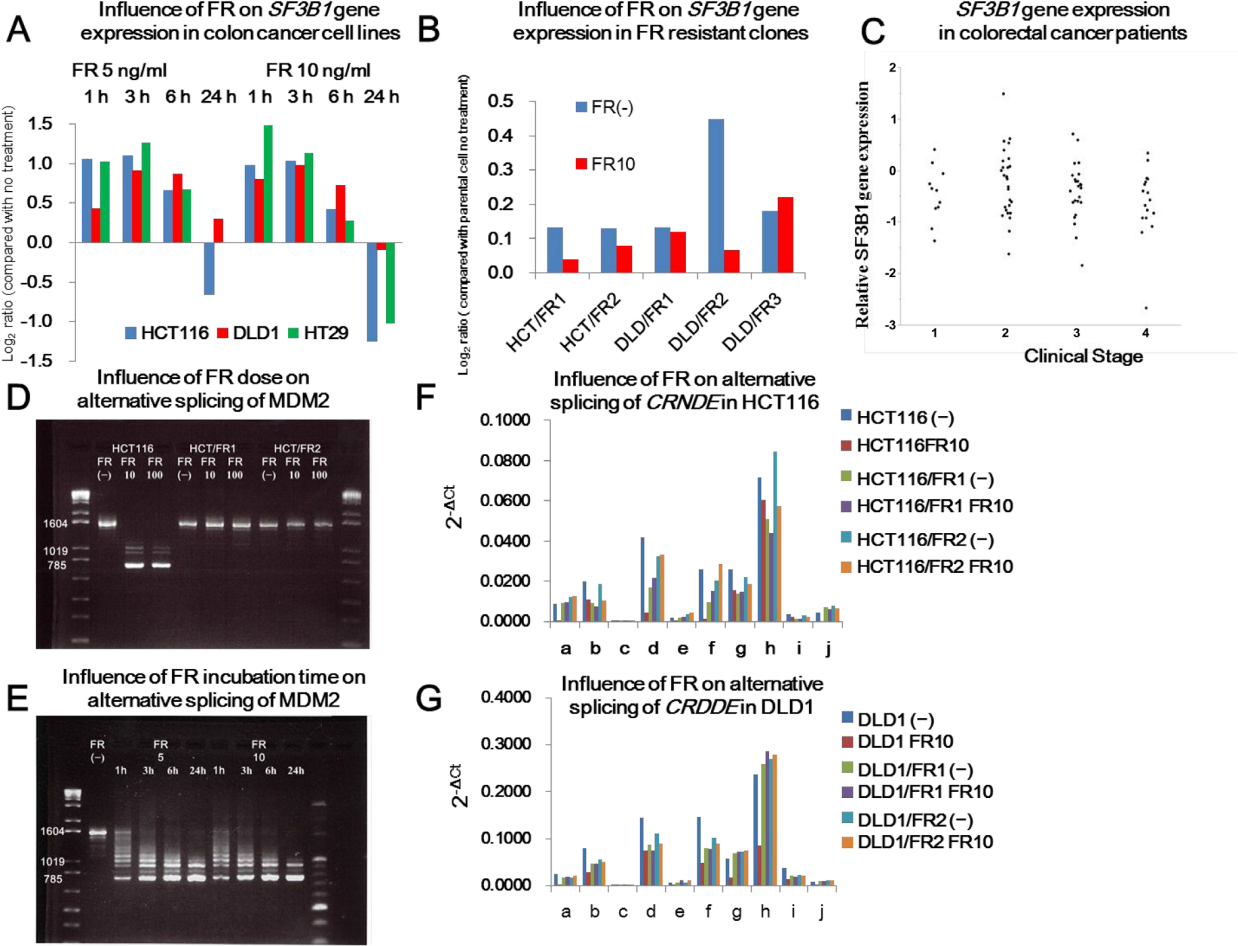
(**A, B**) Influence of FR on *SF3B1* gene expression according to the duration of FR treatment (A) and presence of *SF3B1* mutation (B). A: *SF3B1* expression in HCT116 and HT29 cells was increased by FR treatment for 1–3 h and decreased by further treatment; however, the extent of change was two-fold at most. B: Influence of FR on *SF3B1* gene expression in FR-resistant clones. *SF3B1* expression of FR-resistant clones was unaffected by FR treatment. (**C**) *SF3B1* gene expression in CRC patients. *SF3B1* gene expression in 80 colorectal cancer tissues was compared with that in normal mucosa by real-time quantitative PCR. Relative *SF3B1* expression is shown according to TNM stage. (**D, E**) Influence of FR dose and incubation time on alternative splicing of *MDM2*. FR treatment (10 ng/ml and 100 ng/ml) for 24 h resulted in PCR products of 1019 bp and 785 bp instead of a 1604-bp product due to modulation of alternative splicing of *MDM2* in HCT116 cells but not in FR-resistant clones HCT/FR1 and HCT/FR2 (D). Modulation of alternative splicing was induced by 1 h of FR treatment, resulting in PCR products of sizes other than 1604 bp (E). (**F, G**) Influence of FR on alternative splicing of *CRNDE* in HCT116 and DLD1. FR treatment significantly reduced the expression of variants a, d, f, and j in HCT116 cells, but not in FR-resistant clones HCT/FR1 and HCT/FR2 (F). FR treatment significantly reduced the expression of variants a, b, f, g, h, and j in DLD1 cells, but not in FR-resistant clones DLD/FR1 and DLD/FR2 (G).

FR treatment had a limited effect on *SF3B1* expression in CRC cell lines, with a 2-fold change at most (Figure 2A). FR did not affect *SF3B1* expression in DLD1 cells. However, in HCT116 cells, *SF3B1* expression was enhanced by 1–3 h of FR treatment and inhibited by 24 h of FR treatment. *SF3B1* expression in FR-resistant clones was unaffected by FR treatment, similar to their parental cells (Figure 2B).

We also assessed *SF3B1* expression in cancer tissues compared with normal mucosa (relative *SF3B1* expression) in 80 CRC patients. Relative *SF3B1* expression varied from −2.66 to 1.5, with a median of −0.35 and mean of −0.39 (Figure 2C). There was no significant difference in relative *SF3B1* expression among patients according to clinical stage (I, 10 patients; II, 28 patients; III, 25 patients; IV, 17 patients by TNM classification version 7) [41].

These data suggested that *SF3B1* expression was not greatly influenced by FR treatment, *SF3B1* mutation, or CRC progression.

### Modulation of alternative splicing of *MDM2* and *CRNDE* by FR treatment or *SF3B1* mutation

We next evaluated the effect of FR treatment or *SF3B1* mutation on alternative splicing of *MDM2* (Figure 2D, 2E) and *CRNDE* (Figure 2F, 2G), as previously described [14, 42].

As shown in Figure 2D, FR treatment (10 ng/ml and 100 ng/ml) for 24 h resulted in PCR products of 1019 bp and 785 bp instead of a 1604-bp product as a result of modulation of alternative splicing of *MDM2* in HCT116 cells but not in the FR-resistant clones HCT/FR1 and HCT/FR2 [29]. Modulation of alternative splicing was induced by 1 h of FR treatment, resulting in PCR products of sizes other than 1604 bp (Figure 2E).

*CRNDE*, which possesses 10 transcript variants (a, b, c, d, e, f, g, h, i, j) through alternative splicing, is highly expressed in CRC cell lines and patients [30]. FR treatment significantly reduced the expression of variants a, d, f, and j in HCT116, but not in FR-resistant clones HCT/FR1 and HCT/FR2 (Figure 2F). FR significantly reduced the expression of variants a, b, f, g, h, and j in DLD1 cells, but not in FR-resistant clones DLD/FR1 and DLD/FR2 (Figure 2G). The fact that FR had a different effect on alternative splicing between HCT1116 and DLD1 cells suggests that the influence of FR on alternative splicing would vary among CRCs.

### Pathways affected by SF3B1 inhibition through FR treatment or *SF3B1* mutation

**Figure 3:**
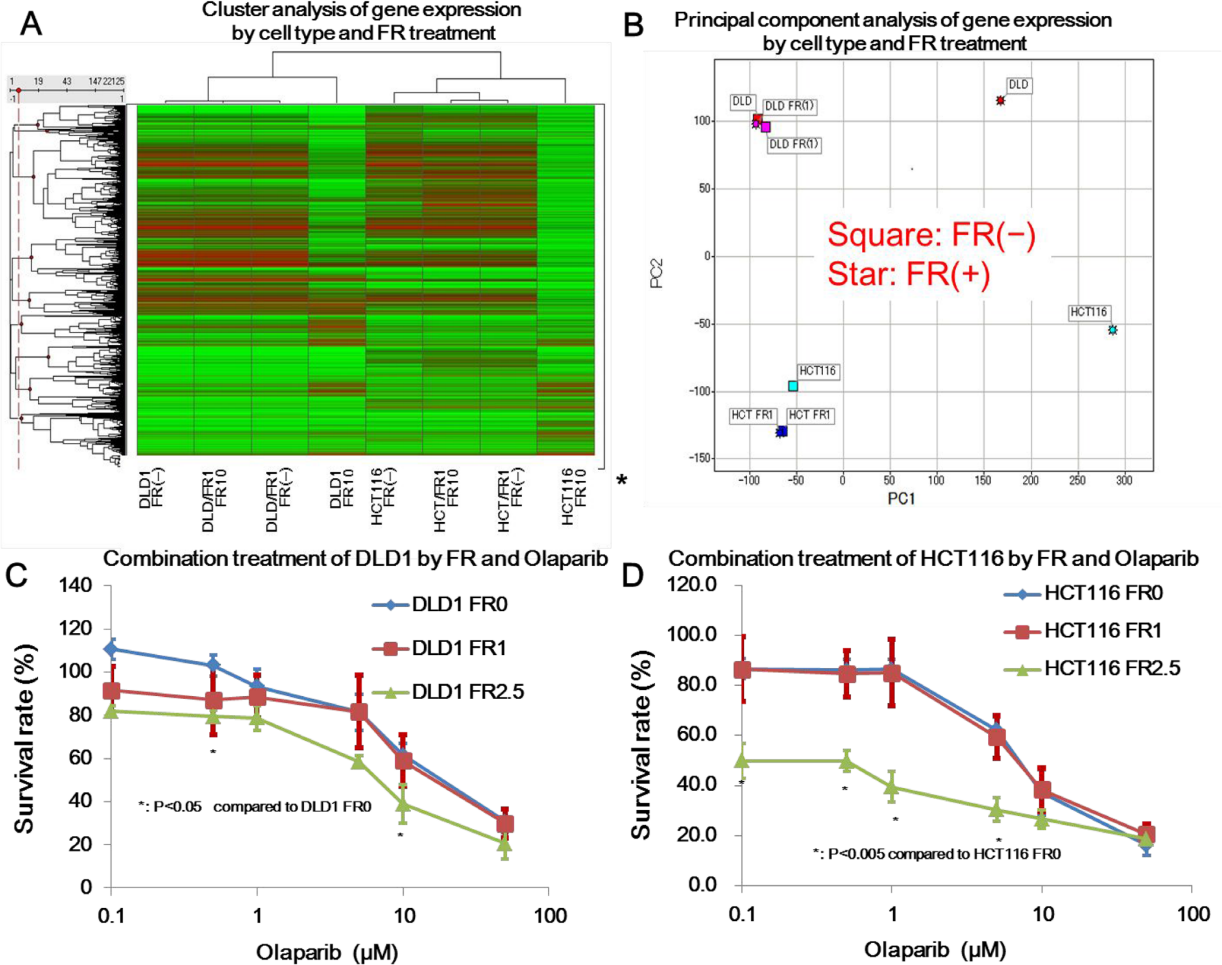
Microarray analysis of gene expression influenced by FR901464 and cytotoxic effect of combination treatment (**A**) Cluster analysis of gene expression by cell type and FR treatment. Cluster analysis was performed based on microarray analysis data by cell type (DLD1, DLD/FR1, HCT116, or HCT/FR1) and FR treatment (no treatment, 10 ng/ml FR for 24 h). Significant differences were observed between with and without treatment. (**B**) Principal component (PC) analysis of gene expression by cell type and FR treatment. Principal component analysis showed that PC1 was associated with FR treatment in both DLD1 and HCT cells, and PC2 was associated with cell type. (**C, D**) Combination treatment of DLD1 and HCT116 with FR and olaparib. In DLD1 cells, pretreatment with FR enhanced cytotoxicity of olaparib (0.5 μM and 10 μM) at an FR concentration of 2.5 ng/ml but not at 1 ng/ml (C). In HCT116 cells, pretreatment with FR enhanced cytotoxicity of olaparib (0.1 μM to 5 μM) at a concentration of 2.5 ng/ml but not at 1 ng/ml (D).

To evaluate the pathways affected FR inhibition of SF3B1 in CRC, we used the Agilent expression array. A total of 22,125 probes were differentially expressed on the basis of signal fold-changes of ≥4 and signal differences of ≥100. Hierarchical cluster analysis indicated significant differences in gene expression patterns by cell type (between DLD1 and HCT116 cells, and between HCT116 and HCT/FR1 cells) and FR treatment (between FR (−) and FR10 in both DLD1 and HCT116 cells) (Figure 3A).

PC analysis also showed significant differences in gene expression by cell type (between DLD1 and HCT116 cells, and between HCT116 and HCT/FR1 cells) and FR treatment (between FR (−) and FR10 in both DLD1 and HCT116 cells) (Figure 3B). In contrast, gene expression in DLD/FR1 and HCT/FR1 cells was not affected by FR treatment. PC1, but not PC2, was associated with FR treatment in both DLD1 and HCT116 cells. PC2 was associated with cell type between DLD1 and HCT116 or between HCT116 and HCT/FR1, although the difference between HCT116 and HCT/FR1 was much smaller than that between DLD1 and HCT116. The cumulative proportion of variance was 47.8% in PC1 and 30.3% in PC2.

We further examined the KEGG pathways according to the results of principal component analysis. A total of 2,691 probes were upregulated ≥2-fold and 4,896 probes were downregulated ≥2-fold in both DLD1 and HCT116 cells. Gene symbols corresponding to these probes showed that 2,474 genes were upregulated, 4,549 were downregulated, and 132 were either upregulated or downregulated depending on the probe. A total of 4,341 genes were differentially expressed in DLD/FR1 or HCT/FR1 compared with their parental cells; of these, 149 genes were upregulated and 30 were downregulated in both DLD/FR1 and HCT/FR1. Therefore, the influence of FR treatment on changes in gene expression was much more consistent in CRC cell lines than in FR-resistant clones.

Pathway analysis predicted the enrichment of several significant pathways. The pathways that were significantly influenced in PC1, which represented differences in FR treatment, were different from those in PC2, which represented differences in cell type (Table 3). These results indicated that FR treatment significantly influenced pathways associated with spliceosomes, the cell cycle, Fanconi anemia, homologous recombination, ubiquitin-mediated proteolysis, nucleotide excision repair, oocyte meiosis, basal transcription factors, and endocytosis. *Cyclin A2*, *Cyclin B1*, *Cyclin E2*, *CDK1*, *CDK6*, and *CDK8* were included among the cell cycle pathway genes affected by FR. *BRCA1*, *BRCA2*, and *PALB2* were among the genes associated with the Fanconi anemia pathway. *BRCA1* and *BRCA2* were also included in the ubiquitin-mediated proteolysis and homologous recombination pathways, respectively.

**Table 3:** Pathways with significant correlations in PC

Rank	Pathway	*P*-value
Pathways with significant positive correlation in PC1		
1	Spliceosome	0.0001
2	Circadian rhythm	0.0027
3	p53 signaling pathway	0.0033
4	mRNA surveillance pathway	0.0049
5	Neuroactive ligand-receptor interaction	0.0065
6	RNA transport	0.0079
Pathways with significant negative correlation in PC1		
1	Cell cycle	0
2	Fanconi anemia pathway	0
3	Ubiquitin mediated proteolysis	0
4	Homologous recombination	0
5	Nucleotide excision repair	0
6	Oocyte meiosis	0
7	Basal transcription factors	0
8	Endocytosis	0
9	SNARE interactions in vesicular transport	0.0001
10	Progesterone-mediated oocyte maturation	0.0001
11	Amino sugar and nucleotide sugar metabolism	0.0003
12	Glycerophospholipid metabolism	0.0017
13	Viral carcinogenesis	0.0022
14	DNA replication	0.0031
15	Phosphatidylinositol signaling system	0.0040
16	Inositol phosphate metabolism	0.0042
17	Pancreatic cancer	0.0042
18	Adherens junction	0.0058
19	HTLV-I infection	0.0070
20	Fructose and mannose metabolism	0.0095
Pathways with significant positive correlation in PC2		
1	Metabolism of xenobiotics by cytochrome P450	0
2	Chemical carcinogenesis	0.0001
3	Drug metabolism - cytochrome P450	0.0001
4	Legionellosis	0.0010
5	Valine, leucine, and isoleucine degradation	0.0012
6	Pentose and glucuronate interconversions	0.0015
7	Glutathione metabolism	0.0028
8	Ascorbate and aldarate metabolism	0.0030
9	TGF-beta signaling pathway	0.0030
10	Pathogenic *Escherichia coli* infection	0.0044
11	Toxoplasmosis	0.0062
12	Pertussis	0.0071
13	Cytokine–cytokine receptor interaction	0.0076
14	Amoebiasis	0.0080
15	Colorectal cancer	0.0085
16	Metabolic pathways	0.0087
Pathways with significant negative correlation in PC2		
1	Type I diabetes mellitus	0
2	Allograft rejection	0.0001
3	Protein digestion and absorption	0.0001
4	Focal adhesion	0.0001
5	PI3K-Akt signaling pathway	0.0002
6	Asthma	0.0003
7	Viral myocarditis	0.0003
8	ECM-receptor interaction	0.0006
9	Graft-versus-host disease	0.0006
10	Cell adhesion molecules (CAMs)	0.0006
11	Axon guidance	0.0009
12	Autoimmune thyroid disease	0.0010
13	Nicotine addiction	0.0010
14	MAPK signaling pathway	0.0011
15	Arginine and proline metabolism	0.0012
16	Inflammatory bowel disease (IBD)	0.0018
17	Glutamatergic synapse	0.0020
18	Proteoglycans in cancer	0.0020
19	Endocytosis	0.0043
20	Calcium signaling pathway	0.0050
21	*Staphylococcus aureus* infection	0.0052
22	Intestinal immune network for IgA production	0.0060
23	Retrograde endocannabinoid signaling	0.0082

### Effect of SF3B1 inhibition and *SF3B1* mutations on driver genes

Of 138 driver genes reported by Vogelstein [43], 52 oncogenes and 60 tumor suppressor genes were affected by FR treatment in either HCT116 or DLD1 cells (Tables 4, 5). Quantitative RT-PCR confirmed the inhibitory effect of FR treatment on 27 oncogenes including *BCL2*, *CTNNB1*, *EGFR*, *ERBB2*, *KRAS*, and *MET* driver genes.

**Table 4:** Oncogenes and cancer-associated genes affected by SF3B1 inhibition by FR

Gene Symbol	Log_2_ ratio compared with no treatment				Gene Symbol	Log_2_ ratio compared with no treatment			
	HCT116	DLD1	HCT116	DLD1		HCT116	DLD1	HCT116	DLD1
	Microarray		qRT-PCR			Microarray		qRT-PCR	
Oncogenes					*MAP2K1*	−0.34	1.25	−6.57	−5.57
*ABL1*	2.92	1.99	−0.82	0.46	*MCL1*	0.36	−0.19	1.68	−0.02
*AKT1*	−0.49	−1.22			*MDM2*	2.42	−0.14	−5.8	−6.4
*AR*	1.40	3.21	-	-	*MDM4*	1.47	1.60	−1.15	0.96
*BCL2*	−1.33	2.04	−5.85	−1.55	*MED12*	−3.14	−2.08	−3.4	−3.13
*BRAF*	−0.82	−1.58	−3.17	−0.69	*MET*	−2.90	−1.67	−5.57	−3.55
*CARD11*	1.67	1.18			*MYC*	1.58	−1.48	−0.28	−3.39
*CBL*	−4.47	−1.76	−4.54	−3.09	*c-MYC*	2.54	0.00	2.33	−0.8
*CCND1*	0.68	−1.43	-	-	*MYCL1*	−3.50	−3.47	−5.7	−6.23
*CRLF2*	1.20	1.12	-	-	*MYCN*	1.61	−2.19	-	-
*CSF1R*	0.73	1.59	-	-	*MYD88*	−2.00	−2.14	−6.72	−4.86
*CTNNB1*	−1.10	−1.00	−1.8	−2.18	*NCOA3*	−3.95	0.32	−4.99	−3.94
*DNMT1*	3.05	−2.11	−2.6	−4.29	*NKX2-1*	−1.68	−0.93	-	-
*DNMT3A*	−2.12	−1.73	−5.8	−5.4	*NRAS*	1.46	1.40	−0.04	−0.14
*EGFR*	−0.53	−1.04	−5.31	−4.54	*PDGFRA*	1.63	0.99	-	-
*ERBB2*	−2.54	−1.07	−3.47	−2.07	*PIK3A*	−2.04	0.84	−3.5	−0.66
*EZH2*	−4.94	−2.72	−5.62	−5.67	PTPN11	−2.49	−0.7	-	-
*FGFR2*	−2.93	−2.06	−4.59	−4.2	*RET*	−1.13	1.87	-	-
*FGFR3*	−3.64	−2.06	−6.75	−4.08	*SETBP1*	0.98	1.98	-	-
*GATA2*	−1.58	−0.41	−3.64	−3.68	*SKP2*	−3.99	−4.29	−4.55	−5.84
*GNA11*	−1.25	−1.72	−3.33	−5.18	*SMO*	−2.83	1.33	−4.56	−1.58
*GNAQ*	−4.40	−0.35	−5.23	−3.29	*SPOP*	−0.25	−1.11	-	-
*HIST1H3B*	−3.63	−3.16	−1.76	−1.78	*SRSF2*	2.79	0.89	1.67	0.84
*HRAS*	1.33	−0.69	−1.18	−1.95	*U2AF1*	−0.75	−1.58	-	-
*IDH1*	−3.11	−1.22	−2.66	−2.16	*VEGF-A*	−2.75	−0.13	−0.26	0.01
*JAK1*	−2.29	−1.48	−2.68	−2.48	Cancer associated genes				
*JAK2*	−1.87	−1.19	−6.34	−6.4	*ALDH1A3*	−5.87	−4.25	−5.89	−6.16
*JAK3*	−1.66	−0.42	−4.2	−1.65	*IGF1R*	−2.19	−0.09	−5.36	−5.7
*Kit*	1.6	−6.3		−3.3	*PALB2*	−3.56	−2.74	−4.06	−3.69
*KRAS*	−3.18	−1.32	−6.57	−5.57	*RAD51*	−6.06	−1.46	−5.88	−5.4
*LMO1*	−3.36	1.02	-	-					

Abbreviations: qRT-PCR; quantitative reverse transcriptase polymerase chain reaction.

**Table 5:** Tumor suppressor genes affected by SF3B1 inhibition by FR

Gene Symbol	Log_2_ ratio compared with no treatment				Gene Symbol	Log_2_ ratio compared with no treatment			
	HCT116	DLD1	HCT116	DLD1		HCT116	DLD1	HCT116	DLD1
	Microarray		qRT-PCR			Microarray		qRT-PCR	
*ACVR1B*	1.35	2.07			*MLL2*	−3.16	−1.97		
*APC*	0.48	1.22			*MLL3*	−0.42	−1.01		
*ARID1A*	0.03	1.26			*MSH2*	−5.23	−2.88		
*ARID2*	−1.38	0.55			*MSH6*	−3.82	1.47		
*ATM*	−1.59	−0.28	−4.35	−3.61	*NF1*	−0.75	−1.62		
*ATRX*	−1.23	0.43			*NF2*	−1.99	−0.78		
*AXIN1*	−1.13	−1.15			*NOTCH1*	−2.90	−1.54	−5.03	−1.93
*B2M*	1.13	0.84			*NOTCH2*	−1.07	−0.97		
*BAP1*	1.27	0.24			*PBRM1*	−1.32	−1.63		
*BCOR*	0.47	1.25			*PHF6*	−2.56	−1.13		
*BRCA1*	−6.10	−3.87	−4.88	−5.15	*PIK3R1*	−2.68	−0.73		
*BRCA2*	−2.94	−3.21	−5.07	−5.55	*PRDM1*	5.38	1.68		
*CASP8*	−2.04	−0.81			*PTCH1*	0.98	2.5		
*CDC73*	0.01	−1.2			*PTEN*	−1.95	−0.81		
*CDKN2A*	0.61	1.81			*RB1*	−4.77	−3.43	−4.4	−6.05
*CDKN2C*	−4.96	−1.9			*RUNX1*	−1.25	0.41		
*CEBPA*	−2.25	−1.26			*SETD2*	0.74	1.77		
*CIC*	0.16	−1.24			*SMAD2*	−3.72	−1.71		
*CREBBP*	0.79	1.98			*SMAD4*	−0.16	1.79		
*CYLD*	−2.3	−0.18			*SMARCA4*	−1.86	−1.44		
*DAXX*	−1.89	−0.54			*SMARCB1*	−3.33	−0.71		
*FAM123B*	1.84	−0.01			*SOCS1*	−1.3	0.58		
*FBXW7*	4.19	1.35			*SOX9*	0.6	−1.18		
*FUBP1*	−3.96	−1.96			*STAG2*	−1.25	−1.08		
*HNF1A*	2.10	1.65			*STK11*	−0.78	−1.12		
*KDM5C*	0.93	1.64			*TET2*	2.89	2.99		
*MAP2K4*	2.12	0.31			*TNFAIP3*	1.84	2.34		
*MAP3K1*	−2.17	−0.01			*TP53*	−1.08	−0.4		
*MEN1*	−1.46	−2.21			*TSC1*	2.66	3.64		
*MLH1*	−3.30	−2.00			*WT1*	1.58	−1.62		

In FR-resistant clones, 36 genes were affected in either DLD/FR1 or HCT/FR1, of which the expression of two genes, *RET* and *SETBP1*, was increased in both clones by microarray (Table 6). In contrast to FR treatment, quantitative RT-PCR did not confirm the changes in gene expression in HCT/FR1 and DLD/FR1 cells bearing *SF3B1* gene mutations (Table 6).

**Table 6:** Driver genes affected by *SF3B1* mutations

Gene symbol	Log_2_ ratio compared with parental cells				Gene symbol	Log_2_ ratio compared with parental cells			
	HCT/FR1	DLD/FR1	HCT/FR1	DLD/FR1		HCT/FR1	DLD/FR1	HCT/FR1	DLD/FR1
	Microarray		qRT-PCR			Microarray		qRT-PCR	
*ABL1*	−1.03	0.03			*NCOA3*	1.08	0.911	−0.04	−0.04
*ARID1B*	−1.27	−0.10			*NCOR1*	1.03	0.00		
*B2M*	1.17	0.09			*NOTCH1*	0.00	1.16	0.55	0.03
*BCL2*	−1.31	0.41	−0.26	−0.12	*PDGFRA*	−0.04	2.77		
*CDH1*	2.16	0.10			*PIK3R1*	1.70	−0.28		
*CDKN2A*	−1.08	−0.12			*PRDM1*	0.69	1.32		
*CSF1R*	−0.36	1.66	−2.07	3.43	*PTCH1*	1.32	0.02		
*DNMT3A*	1.22	0.25	1.34	−0.84	*RET*	1.01	1.13	0.56	ND
*ERBB2*	1.22	0.35	1.35	0.08	*RNF43*	−7.04	−0.32		
*FGFR2*	1.37	0.17	1.7	−0.49	*RUNX1*	−3.10	−0.26		
*FGFR3*	1.06	0.17	0.75	−1.34	*SETBP1*	2.01	2.07		
*GATA2*	2.02	0.46	1.97	0.28	*SKP2*	−1.02	0.02	−0.02	−0.45
*GNAS*	−1.86	1.47	0.63	−0.47	*SMO*	0.08	1.08	0.61	0.10
*HNF1A*	0.11	1.26			*SOCS1*	−1.05	0.61		
*IDH1*	1.25	0.00	1.56	−0.25	*STAG2*	−0.20	−1.37		
*KDM6A*	1.29	0.05			*TNFAIP3*	−1.28	0.17		
*KLF4*	1.85	−0.34			*TSC1*	0.56	1.22		
*MYC*	−1.56	−0.06	−1.02	−0.33	*WT1*	−0.03	−2.15		

### Effect of SF3B1 inhibition on Fanconi anemia-associated genes and cancer-associated genes

qRT-PCR data confirmed that the expression of Fanconi anemia-associated genes including *BRCA1*, *BRCA2*, *PALB2*, and *RAD51* and cancer-associated genes (*ALDH1A3* and *IGF1R*) was strongly inhibited by FR treatment, as shown by microarray analysis (Table 3). Inhibition of *BRCA1* and *BRCA2* gene expression suggested a synergistic cytotoxic effect with PARP1 inhibitor based on previous findings in breast cancer with mutation of *BRCA1* or *BRCA2* [44]. *PALB2*, *ALDH1A3*, and *IGF1R* were reported to show associations with sensitivity to mitomycin, stem cell markers, and cancer progression, respectively [45–47].

### Combination treatment with FR and olaparib

Based on the above findings of inhibition of *BRCA1* and *BRCA2* expression by FR, we assessed the effect of combination treatment with FR and olaparib. In DLD1 cells, pretreatment with FR enhanced cytotoxicity of olaparib (0.5 μM and 10 μM: *P* < 0.05) at a FR concentration of 2.5 ng/ml but not at 1 ng/ml (Figure 3C). In HCT116 cells, pretreatment with FR enhanced the cytotoxicity of olaparib (0.1 μM to 5 μM: *P* < 0.005) at 2.5 ng/ml but not at 1 ng/ml. (Figure 3D). These findings are consistent with the data of combination index (CI). In DLD1 cells, CI was 0.84 and 1.08 at a FR concentration of 2.5 ng/ml and 1 ng/ml, respectively. In HCT116 cells, CI was 0.93 and 1.37 at a FR concentration of 2.5 ng/ml and 1 ng/ml, respectively. Synergistic effects were shown at a FR concentration of 2.5 ng/m in both DLD1 and HCT116.

## DISCUSSION

In this study, we assessed the possible application of the splicing modulator FR for CRC treatment. FR showed effective cytotoxicity against CRC cells *in vitro* and *in vivo.* This is the first report to show the efficacy of FR analogues in solid tumors, although the antitumor effect was dependent on the cell line and was associated with severe toxicity *in vivo*. The instability of FR under serum-containing conditions has been described [5, 9, 11]. FR analogues including SSA and Meayamycin appear to be more stable than FR in serum [5, 18]; however, sufficient efficacy of FR analogues for solid tumors has not yet been demonstrated [18, 21, 22]. Although we considered that intraperitoneal administration of FR might reduce both toxicity and efficacy *in vivo*, FR via this route maintained both toxicity and efficacy. Therefore, improvement of drug delivery to reduce toxicity is indispensable for monotherapy.

All of the FR-resistant clones carried mutations in codon 1074 of the *SF3B1* gene, which is consistent with a previous report on another SF3B1 inhibitor, Pladienolide [16, 23]. In contrast, DLD1, COLO829, and HCC38 cells, which contained mutations at other sites in the *SF3B1* gene, were sensitive to FR. Therefore, mutation sites associated with FR sensitivity are different from those associated with cancer progression. Our study also showed low frequencies of *SF3B1* gene mutations and no mutations in codon 1074 of the *SF3B1* gene in CRC patient samples. Thus, these results indicate that CRC cells, and possibly all cancer cells, are intrinsically sensitive to splicing modulators.

The influence of FR on alternative splicing of *MDM2* and *CRNDE* as a result of splicing modulation was abrogated in these FR-resistant clones. Moreover, the effect of FR on gene expression, including suppression of oncogenes and cancer-associated genes, was also abolished in FR-resistant clones. These results indicated that the cytotoxicity of FR was associated with its influence on gene splicing and gene expression through inhibition of SF3B1 function.

Splicing modulators have been reported to inhibit expression of VEGF, c-MYC, and MCL-1 [12, 22, 48]. A previous study reported downregulation of VEGF and MCL-1 and upregulation of c-MYC by a splicing modulator. In our microarray study, downregulation of *VEGFA* in HCT116 and upregulation of *MYC* in both DLD1 and HCT116 were the same as the previous study. However, *MCL1* was upregulated in DLD1 and does not reflect the findings of the previous report. Differences from previous studies may depend on variations in cell types and evaluation methods.

Our study suggested that splicing modulation might downregulate many oncogenes and cancer-associated genes. Moreover, splicing modulators could influence gene expression associated with drug sensitivity. Inhibition of *BRCA1* and *BRCA2* expression by FR suggested a synergistic cytotoxic effect with olaparib based on the fact that this PARP1 inhibitor is effective in breast cancer with genetic deficiencies in *BRCA1* or *BRCA2* [44]. The enhanced cytotoxicity of olaparib after pretreatment with FR in this study supports this hypothesis. Combination therapy with splicing modulators to overcome drug resistance has been reported in lung cancer cells and melanoma [17, 21].

Our study showed low frequencies of *SF3B1* mutations and no mutations in codon 1074 of the *SF3B1* gene in CRC patient samples. These results indicate that CRC cells, and possibly all cancer cells, are intrinsically sensitive to splicing modulators.

*SF3B1* expression levels in the CRC patients of our study were not associated with tumor progression, in contrast to previous findings [48, 49]. This discrepancy may reflect differences in sample conditions; we stored samples for RNA isolation in RNAlater at −80°C whereas the other studies collected RNA from samples in which the RNA might be fragmented.

Our study has several limitations. First, the severe toxicity of FR in the xenograft model prevented us from further examining the efficacy of FR *in vivo*. However, the lower IC_50_ value of FR in RKO cells *in vitro* seemed to be associated with efficacy *in vivo*. Improved FR delivery *in vivo* is critical for sufficient efficacy and reduced toxicity. Second, we did not show that mutations in codon 1074 of *SF3B1* definitively induced FR resistance, although the presence of codon 1074 mutations in all the FR-resistant clones, in pladienolide-resistant clones, and in clones derived from CT26 cells with MSS strongly implicates a role for mutations in codon 1074 of *SF3B1* in FR resistance. Third, the influence of FR on driver oncogenes and cancer-associated genes was not evaluated in other types of cancer and with other splicing modulators. A consistent effect on driver oncogenes and cancer-associated genes, regardless of cancer type and splicing modulator, will promote further study of splicing modulators. Finally, the effect of combination therapy was not confirmed *in vivo* because of a shortage of FR.

In conclusion, our study indicates the possible application of splicing modulators in combination therapy for the treatment of cancers including CRC through their influence on gene expression associated with drug sensitivity.

## MATERIALS AND METHODS

### Drugs and chemicals

FR, a gift from Astellas Pharma Inc. (Tokyo, Japan), and olaparib (Funakoshi, Tokyo, Japan) were dissolved in DMSO at a concentration of 100 μg/ml and stored at −20°C. OHP (Yakult Inc., Tokyo, Japan) was stored at 4°C. 5-FU (Sigma-Aldrich Japan, Tokyo, Japan) was dissolved in phosphate-buffered saline at a concentration of 10 mM and stored at 4°C. All drugs were diluted in culture medium immediately before use.

### Cell lines and cloning of drug-resistant cells

The human melanoma cell line COLO829, human breast cancer cell line HCC38, and murine colon tumor cell line CT26 were purchased from ATCC (Manassas, VA, USA). Human CRC cell lines Caco2, DLD1, HCT116, LoVo, HT29, and RKO were gifts from Dr. Hirofumi Yamamoto (Osaka University Graduate School of Medicine, Japan). All human cell lines were authenticated by ATCC using DNA profiling. Cells were maintained in appropriate media as follows: DMEM (DLD1 and HCT116), RPMI (HT29, RKO, COLO829, CT26, and HCC38), and Hanks-F12K (LoVo) supplemented with 10% fetal bovine serum, 10,000 units penicillin, 10 mg/ml streptomycin, and 25 μg/ml amphotericin B. Culture media and fetal bovine serum were obtained from Life Technologies Japan (Tokyo, Japan). All cells were grown at 37°C in a humidified incubator with 5% CO_2_. To obtain FR-resistant cells, cells were cultured in the presence of high concentrations of FR (20 ng/ml for DLD1 and 10 ng/ml for all other cell lines).

### Patients

Specimens were collected from 318 patients who underwent surgery at our department. All protocols were approved by the ethics committee of Hyogo College of Medicine as RINHI-0120 and all patients provided written informed consent. The specimens consisted of 312 CRC samples (299 primary tumors and 13 metastatic tumors), two small bowel gastrointestinal stromal tumors (GISTs), and one case each of small intestinal adenocarcinoma, rectal carcinoid, retroperitoneal liposarcoma, and metastatic renal cancer in the colon. The specimens were obtained with adjacent normal tissues for comparison and stored at −80°C before use. For RNA analysis, samples were stored with RNAlater (Qiagen, Venlo, Netherlands).

### Drug treatment in cell culture

Cells were seeded in 200 μl medium in 96-well flat-bottom plates at a density of 2×10^3^ cells per well. The next day, the medium was removed and various concentrations of FR (0.1−500 ng/ml), OHP (0.1−500 μM), and 5-FU (0.1−500 μM) were added. To assess the time dependency, cells were incubated with FR for 1 h, 3 h, 6 h, 12 h, and 96 h. To assess the effect of combination treatment with FR and olaparib, cells were treated with FR (0, 1 ng/ml, 2.5 ng/ml) for 1 h followed by treatment with olaparib at various concentrations for 96 h. After drug treatment, cells were counted using a cell counting kit (Dojindo Laboratories, Kumamoto, Japan) according to the manufacturer’s instructions. Half maximal inhibitory concentration (IC_50_) was calculated as the concentration that corresponded to a 50% reduction in cellular proliferation compared with untreated cells. Experiments were independently performed at least three times and data are shown as means ± standard deviations.

### Mutation analysis of *SF3B1* by Sanger sequencing

Genomic DNA was extracted from eight cancer cell lines (six CRC cell lines, COLO829, and HCC38), nine FR-resistant clones, and 253 patient-derived malignant tumors (including 246 CRC patients) using the QIAamp DNA mini kit (Qiagen) (Table 2). We analyzed all 25 exons of *SF3B1* in all cancer cell lines and FR-resistant clones in addition to 81 tumors, including 75 CRC patients, by Sanger sequencing as previously described [26]. Based on these findings, we further examined specific *SF3B1* exons in the remaining 172 tumors, including 171 CRCs (Table 2).

### Microsatellite instability

Genomic DNA from patients was used to evaluate their microsatellite instability (MSI) status. The mononucleotide microsatellite markers BAT-25, BAT-26, NR21, NR22, and NR24 were used for evaluation as previously described [50].

### Human tumor xenografts

Five-week-old female BALB/cAJcl-/nu/nu mice were purchased from Japan Clea Inc. (Tokyo, Japan). Mouse care and experiments were performed under specific pathogen-free conditions. All animal protocols were approved by the Institutional Animal Care and Use Committee of Hyogo College of Medicine and performed at the Institute of Experimental Animal Sciences of Hyogo College of Medicine.

A total of 5×10^6^ HCT116 or RKO cells were subcutaneously injected into mice and the resultant tumors were allowed to grow larger than 5 mm in diameter. FR (0.5 mg/kg or 0.75 mg/kg) was injected intraperitoneally every fourth day into seven mice that received HCT116 cells and nine mice that received RKO cells for a total of six treatments. Tumor volume was calculated as a × b^2^, where a represents the tumor length and b represents its width.

### Microarray

A total of 1×10^6^ DLD1, HCT116, DLD/FR1, or HCT/FR1 cells were seeded into P10 tissue culture plates. After overnight culture, the cells were incubated for a further 24 h with or without 10 ng/ml FR and RNA was extracted using the TRIzol RNA Purification kit (Invitrogen, Waltham, MA, USA). Gene expression profiles were analyzed using the Agilent SurePrint G3 Human GE 8x60K v2 Microarray kit (Agilent Technologies, Santa Clara, CA, USA) according to the manufacturers’ instructions at Dragon Genomic Center (Mie, Japan). The data set is available at Gene Expression Ominibus under accession number GSE58241.

### Microarray data analysis

Signal data were imported into GeneSpring (Agilent) for analysis. Among the 40,380 probes evaluated to be suitable, 22,125 were extracted as differentially expressed genes based on signal fold-changes of ≥4 and signal differences of ≥100. Hierarchical analysis and principal component (PC) analysis were performed to explore similarities and differences in gene expression following FR treatment and in the presence of *SF3B1* mutations. Based on the principal analysis, probes in the first and second principal component were used for further Kyoto Encyclopedia of Genes and Genomes (KEGG) pathway analysis.

### PCR and quantitative real-time reverse transcription PCR (qRT-PCR)

Total RNA was extracted from CRC cell lines and samples from 80 CRC patients using an RNeasy Mini kit (Qiagen). CRC samples stored with RNAlater were used for qRT-PCR. After generation of cDNA using Superscript III reverse transcriptase (Invitrogen), PCR was performed using specific oligonucleotides (Supplementary Table 1) to quantify gene expression and for RNA sequencing of *SF3B1.* Primers for *SF3B1* sequencing have been reported previously [26].

To assess *MDM2* RNA splicing, products were analyzed by agarose gel electrophoresis and ethidium bromide staining [14]. qRT-PCR was performed using the Power SYBR Green Master Mix and appropriate primer pairs (Life Technologies, Supplementary Table 1). Data were collected from wells in triplicate and each experiment was performed at least twice. ^ΔΔ^CT was calculated by evaluating the difference in ^∆^CT between the target gene and *GAPDH* as a control. The methods used to assess variants of *CRNDE* were reported previously [42].

### Statistical analysis

Tukey-Kramer test was used to evaluate the IC_50_ of FR in cell lines. Dunnett’s test was used to evaluate time- and dose-dependency of FR with 12 h treatment as the control. Dunnett’s test was also used to assess the IC_50_ of FR, OHP, and 5-FU in FR-resistant clones relative to the parental cells. The efficacy of combination treatment of FR and olaparib was assessed by Dunnett’s test relative to treatment without FR. Student’s *t*-test was used to evaluate tumor volume in xenograft models. *P*-values less than 0.05 were considered statistically significant. To evaluate the effect of combination therapy of FR and olaparib, combination index (CI) was calculated [51]. A CI of less than 1 was considered to be a synergistic effect.

## SUPPLEMENTARY MATERIALS TABLE



## Abbreviations

CLL: chronic lymphocytic leukemia
CRC: colorectal cancer
CRNDE: colorectal neoplasia differentially expressed
EGFR: epidermal growth factor receptor
5-FU: 5-fluorouracil
FR: FR901464
GIST: gastrointestinal stromal tumors
HDACI: histone deacetylase inhibitor
KEGG: Kyoto Encyclopedia of Genes and Genomes
IC_50_: half maximal inhibitory concentration
MDS: myelodysplastic syndrome
MSI: microsatellite instability
MSS: microsatellite stability
OHP: oxaliplatin
PC: principal component
SF3B1: splicing factor 3B subunit 1
VEGF: vascular endothelial growth factor
