# Supplementary material for: Splicing modulator FR901464 is a potential agent for colorectal cancer in combination therapy
Source: Oncotarget. 2019 Jan 8;10(3):352–67. doi: 10.18632/oncotarget.26564 (PMC6349454; doi:10.18632/oncotarget.26564)
Supplement: Supplementary file 1 [file oncotarget-10-352-s001.pdf]

Splicing modulator FR901464 is a potential agent for colorectal cancer in combination therapy

SUPPLEMENTARY MATERIALS

Supplementary Table 1: Primers used for analysis

Gene symbol	Forward (F) or Reverse (R)	Sequence (5′–3′)
ABL1	F	AAGTCAGATGCTACTGGCCG
ABL1	R	GGAGCAGGGAAGAAGGAATC
ATM	F	TTCAAAGGATTCATGGTCCAG
ATM	R	GCTGTGAGAAAACCATGGAA
BCL2	F	ATGTGTGTGGAGAGCGTCAACC
BCL2	R	TGAGCAGAGTCTTCAGAGACAGCC
BRAF	F	GTGGATTATGCTCCCCACC
BRAF	R	CTGCCATTCCGGAGGAG
BRCA1	F	GAATGGATGGTACAGCTGTGTG
BRCA1	R	ATGGAAGCCATTGTCCTCTGTC
BRCA2	F	GCCACTTTCAAGAGACATTCAACA
BRCA2	R	GTACAGTCTTTAGTTGGGGTGGA
CBL	F	ATGTGGCTGAAGATGAGGGA
CBL	R	AAGCCTCTTCAAGGAGGGAA
CSF1R	F	TTGGGCTGATCCTCTTCTTC
CSF1R	R	AAAGCGTGAGAGCACGAAGT
CTNNB1	F	ATTGTCCACGCTGGATTTTC
CTNNB1	R	TCGAGGACGGTCGGACT
DNMT1	F	TTCTGTTAAGCTGTCTCTTTCCA
DNMT1	R	TGCTGAAGCCTCCGAGAT
DNMT3A	F	ATTCCTTCTCACAACCCGC
DNMT3A	R	TACTTCCAGAGCTTCAGGGC
EGFR	F	TCCTCTGGAGGCTGAGAAAA
EGFR	R	GGGCTCTGGAGGAAAAGAAA
ERBB2	F	AGCATGTCCAGGTGGGTCT
ERBB2	R	CTCCTCCTCGCCCTCTTG
EZH2	F	AATCAGAGTACATGCGACTGAGA
EZH2	R	GCTGTATCCTTCGCTGTTTCC
FGFR2	F	ACCTCTAGCGACTCCCCTG
FGFR2	R	CGGCCCTCCTTCAGTTTAGT
FGFR3	F	TCAGCTCCACAGCATCCC
FGFR3	R	GTCCTTGGGGACGGAGC
GAPDH	F	TGCACCACCAACTGCTTAGC
GAPDH	R	GGCATGGACTGTGGTCATGAG
GATA2	F	ACAATTTGCACAACAGGTGC
GATA2	R	CACAAGATGAATGGGCAGAA
GNA11	F	GCATCTGCTTGATGAACGTG
GNA11	R	TGAGGTGAAGGAGTCCAAGC
GNAQ	F	ACGTACTCTTGCCACTCTCTCC
GNAQ	R	GGATCAACGACGAGATCGAG
GNAS	F	TTCCAAAAAGGGACCCATC
GNAS	R	TTCCAAAAAGGGACCCATC
HIST1H3B	F	AAGTGGGTGGCTCTGAAAAG

HIST1H3B	R	CATCCATGCTAAGCGAGTGA
HRAS	F	CCAGCTTATATTCCGTCATCG
HRAS	R	CAGTCGCGCCTGTGAAC
IDH1	F	CTTTTGGGTTCCGTCACTTG
IDH1	R	GTCGTCATGCTTATGGGGAT
JAK1	F	GAATGACGCCACACTGACTG
JAK1	R	GATGACAAGATGTCCCTCCG
JAK2	F	CCATTCCCATGCAGAGTCTT
JAK2	R	CAGGCAACAGGAACAAGATG
JAK3	F	CAGCCTCCGTGGACAAGAG
JAK3	R	CTTCGAAAGTCCAGGGTCC
KIT	F	TGATTTTCCTGGATGGATGG
KIT	R	TGGGATTTTCTCTGCGTTCT
KRAS	F	TGACCTGCTGTGTCGAGAAT
KRAS	R	TTGTGGACGAATATGATCCAA
MDM2	F	CTGATCCAACCAATCACCTG
MDM2	R	AAGCCTGGCTCTGTGTGTAA
MDM4	F	TGAACACTGAGCAGAGGTGG
MDM4	R	GGGAGCGACTCATGGAGC
MED12	F	GGGATCTTGAGCTACGAACAC
MED12	R	GCAGGCTGGTTATTGAAACCTTG
MET	F	TGTTCGATATTCATCACGGC
MET	R	GCATTTTTACGGACCCAATC
MYC	F	CACCGAGTCGTAGTCGAGGT
MYC	R	TTTCGGGTAGTGGAAAACCA
MYCL1	F	GTCGTAGAAATAGTGCTGGTACG
MYCL1	R	GTGCGTGTGTGCTGGCT
MYD88	F	GCCACCTGTAAAGGCTTCTC
MYD88	R	GACTGCTCGAGCTGCTTACC
NCOA3	F	CAGTCAAAGGATGTTCAAGCA
NCOA3	R	CCGATTTAAAGCTGAGCTGC
NOTCH1	F	GTTGGGGTCCTGGCATC
NOTCH1	R	GGTGAGACCTGCCTGAATG
NRAS	F	GCACCATAGGTACATCATCCG
NRAS	R	GCTTCCTCTGTGTATTTGCCA
PALB2	F	TGTGATGCTGTACTGTCTTCCTC
PALB2	R	GCAATTGTTCCAGAAGTCAAGAT
PIK3CA	F	CGTGGAGGCATTGTTCTGAT
PIK3CA	R	GGAGCCTGGAAGAGCCC
RAD51	F	TGTTTGGAGAATTCCGAACTG
RAD51	R	GTCAATGTACATGGCCTTTCCT
RB1	F	CCTTCTCGGTCCTTTGATTG
RB1	R	CAGAAGGCAACTTGACAAGAGA
RET	F	AAGCATCCCTCGAGAAGTAGA
RET	R	CTAGCCGCAGTCCCTCC
SF3B1	F	TATTGCTGACAGGGGAGCTG
SF3B1	R	AATACATCATGAGGGCCAATG
SKP2	F	GAAGGGAGTCCCATGAAACA
SKP2	R	GCTGAAGAGCAAAGGGAGTG
SMO	F	GGTCATTCTCACACTTGGGC
SMO	R	GGACTCCCAGGAGGAAGC
SRSF2	F	CCACTCAGAGCTATGAGCTACG
SRSF2	R	ACTCCTTGGTGTAGCGGTCC
